# Supplementary figures and images for: Copy-back viral genomes induce a cellular stress response that interferes with viral protein expression without affecting antiviral immunity
Source: PLoS Biol. 2023 Nov 20;21(11):e3002381. doi: 10.1371/journal.pbio.3002381 (PMC10695362; doi:10.1371/journal.pbio.3002381)

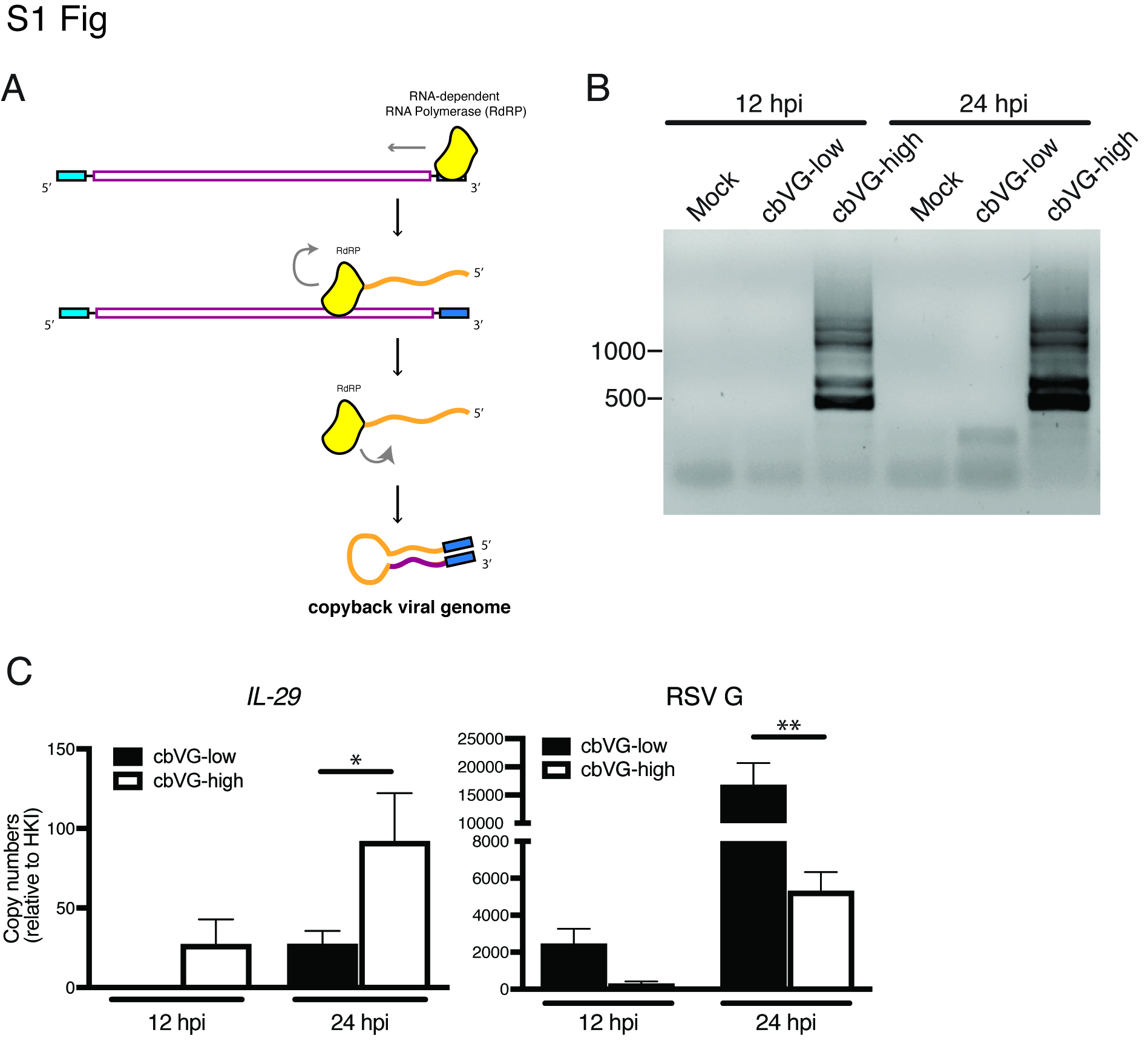

Supplement: S1 Fig — (A) Diagram showing how cbVGs form during negative-sense single-stranded RNA virus infection. (B) Agarose gel of cbVG PCR amplicons from A549 cells 24 hpi with RSV cbVG-high virus at MOI 1.5 TCID50/cell. (C) Expression of RSV G and IL-29 mRNAs in A549 cells 24 hpi with RSV cbVG-high or cbVG-low virus at MOI 1.5 TCID50/cell. Statistical analysis: one-way ANOVA (*p < 0.05, **p < 0.01). Numerical values plotted can be found in the Supporting information: S1 Data. cbVG, copy-back viral genome; hpi, hours postinfection; MOI, multiplicity of infection; RSV, respiratory syncytial virus. (TIF) [file pbio.3002381.s001.tif]

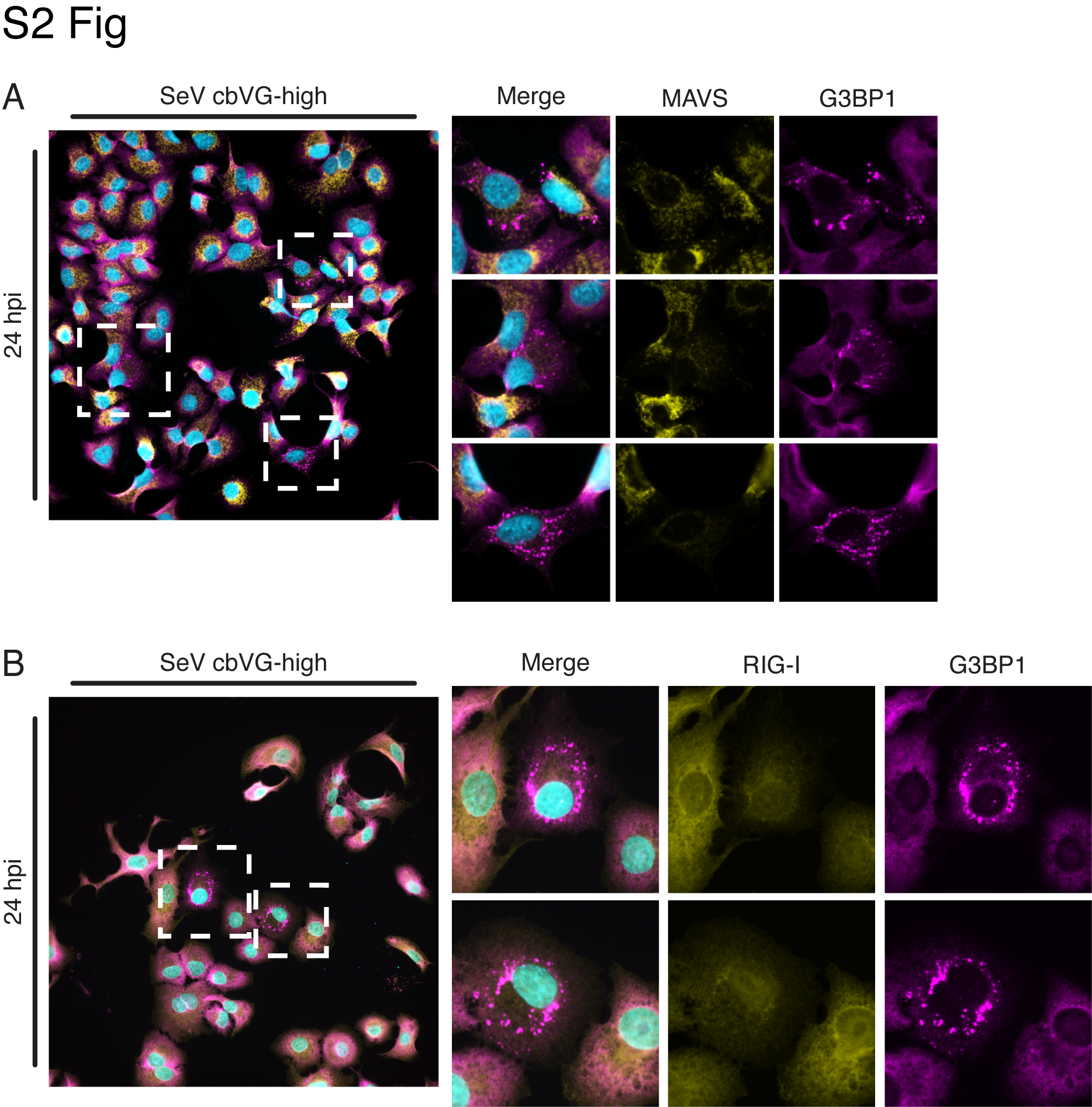

Supplement: S2 Fig — (A) SG (G3BP1, magenta) and MAVS (yellow) staining in A549 cells 24 hpi with RSV cbVG-high virus MOI 1.5 TCID50/cell. Zoomed in images of SG-positive cells are shown on the right with merge and MAVS and G3BP1 single channel. (B) SG (G3BP1, magenta) and RIG-I (yellow) staining in A549 cells 24 hpi with RSV cbVG-high virus MOI 1.5 TCID50/cell. Zoomed in images of SG-positive cells are shown on the right with merge and RIG-I and G3BP1 single channel. Widefield images at 40× magnification. cbVG, copy-back viral genome; G3BP1, GTPase-activating protein-binding protein 1; hpi, hours postinfection; MAVS, mitochondrial antiviral signaling; MOI, multiplicity of infection; RIG-I, retinoic acid–inducible gene I; RSV, respiratory syncytial virus; SeV, Sendai virus; SG, stress granule. (TIF) [file pbio.3002381.s002.tif]

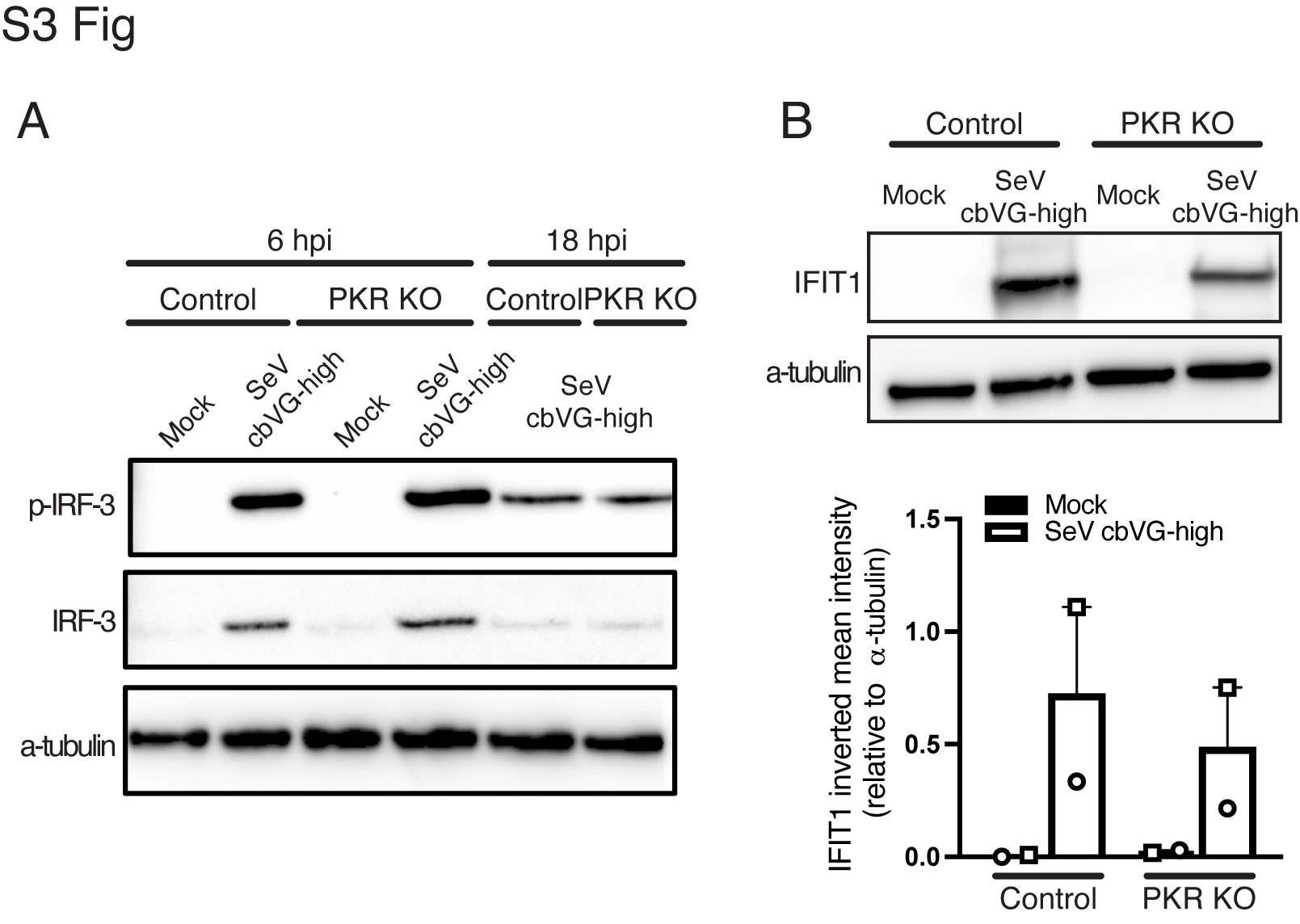

Supplement: S3 Fig — (A) Western blot analysis of phosphorylated IRF-3 in control and PKR KO cells 6 and 18 hpi with SeV cbVG-high at MOI 1.5 TCID50/cell. (B) Western blot analysis of IFIT1 in A549 control and PKR KO cells 24 hpi with SeV cbVG-high at MOI 1.5 TCID50/cell. IFIT1 inverted mean intensity relative to α-tubulin is shown. Images shown are representative of 2 independent experiments. Numerical values plotted can be found in the Supporting information: S1 Data. cbVG, copy-back viral genome; hpi, hours postinfection; KO, knockout; MOI, multiplicity of infection; SeV, Sendai virus. (TIF) [file pbio.3002381.s003.tif]

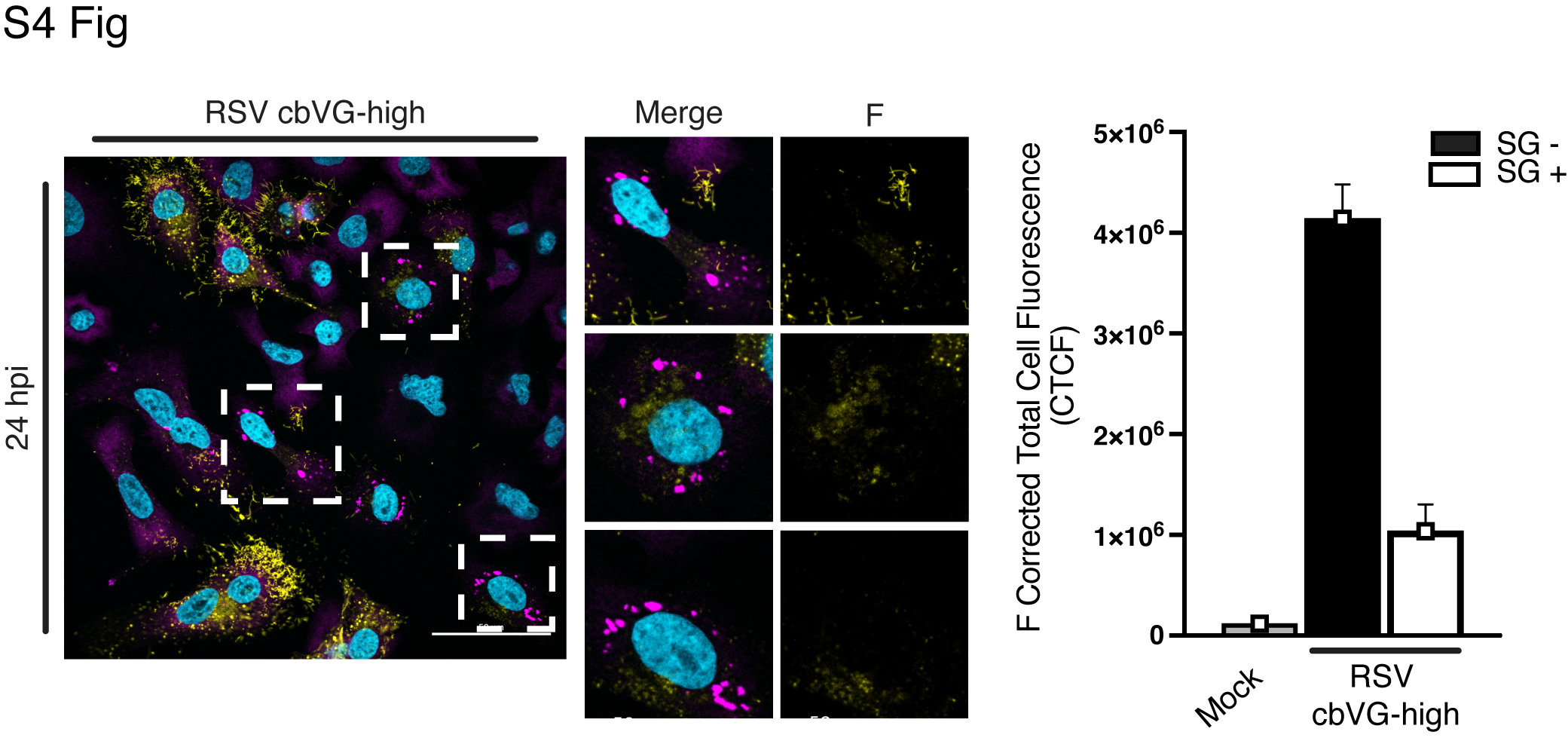

Supplement: S4 Fig — SG (G3BP1, magenta) and viral protein (RSV F, yellow) detection in A549 cells 24 hpi with RSV cbVG-high virus at MOI 1.5 TCID50/cell. Zoomed in images of SG-positive cells are shown on the right with merge and RSV F single channel. Widefield image was acquired with the Apotome 2.0 at 63× magnification, scale bar = 50 μm. Measurements of CTCF are shown on the right. Data points represent the average of approximately 50 cells per group. Numerical values plotted can be found in the Supporting information: S1 Data. cbVG, copy-back viral genome; CTCF, corrected total cell fluorescence; G3BP1, GTPase-activating protein-binding protein 1; hpi, hours postinfection; MOI, multiplicity of infection; RSV, respiratory syncytial virus; SG, stress granule. (TIF) [file pbio.3002381.s004.tif]

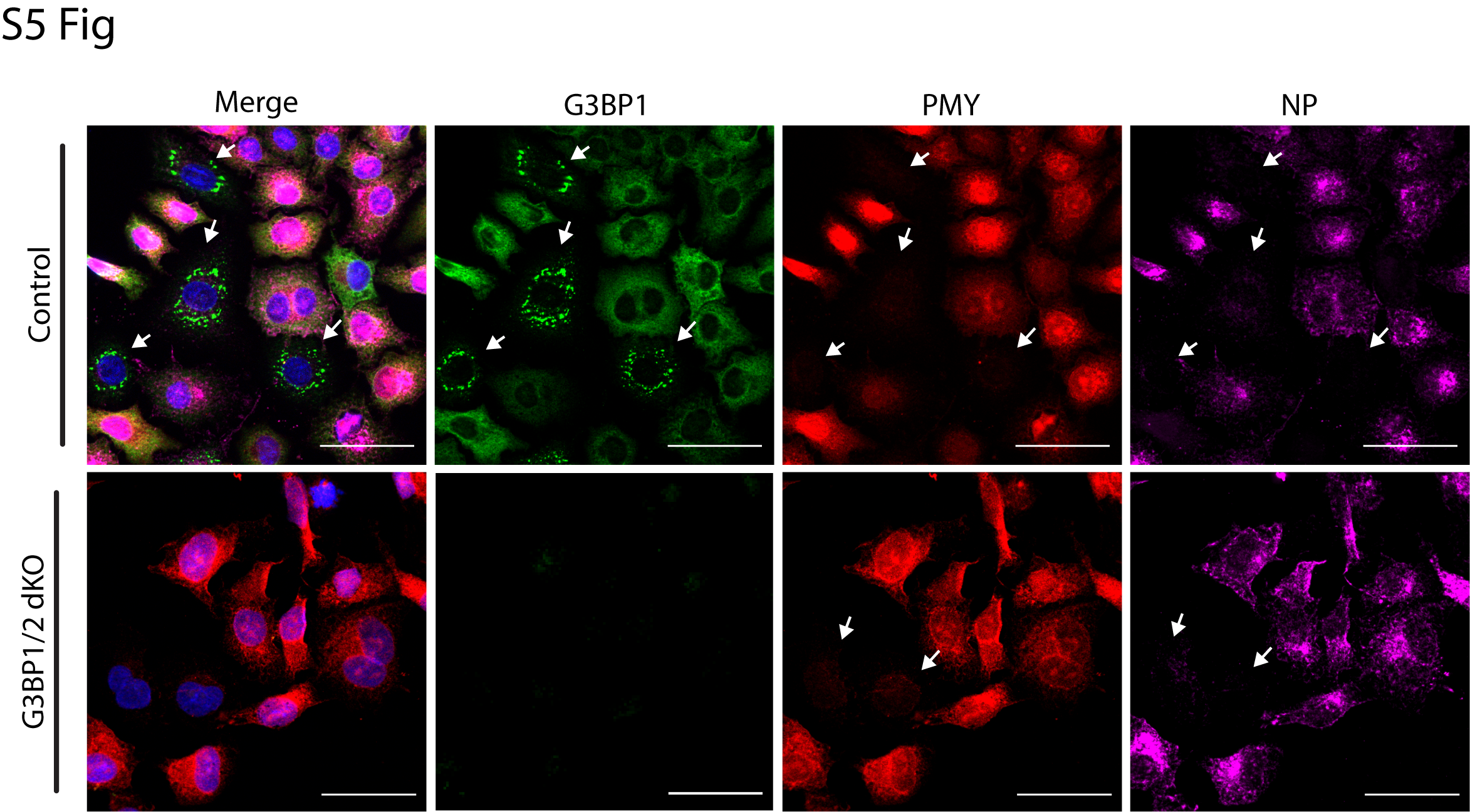

Supplement: S5 Fig — G3BP1 (green) for SG detection and PMY (red) for translation in A549 control and G3BP1/2 dKO cells infected with SeV cbVG-high (SeV NP, magenta) at MOI 3 TCID50/cell 24 hpi. cbVG, copy-back viral genome; dKO, double KO; G3BP1, GTPase-activating protein-binding protein 1; hpi, hours postinfection; MOI, multiplicity of infection; NP, nucleoprotein; PMY, puromycin; SeV, Sendai virus; SG, stress granule. (TIF) [file pbio.3002381.s005.tif]
