# Supplementary material for: Copy-back viral genomes induce a cellular stress response that interferes with viral protein expression without affecting antiviral immunity
Source: PLoS Biol. 2023 Nov 20;21(11):e3002381. doi: 10.1371/journal.pbio.3002381 (PMC10695362; doi:10.1371/journal.pbio.3002381)
Supplement: S1 Raw Images — PDF file containing all original blots and gels used in the main and supporting figures of the manuscript. (PDF) [file pbio.3002381.s009.pdf]

Fig 4A

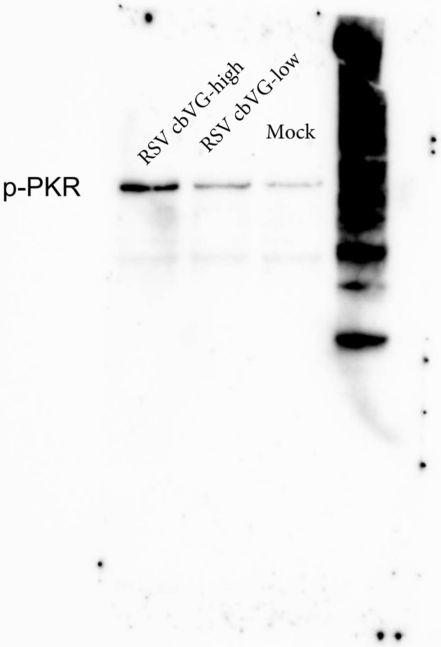

Fig 4A

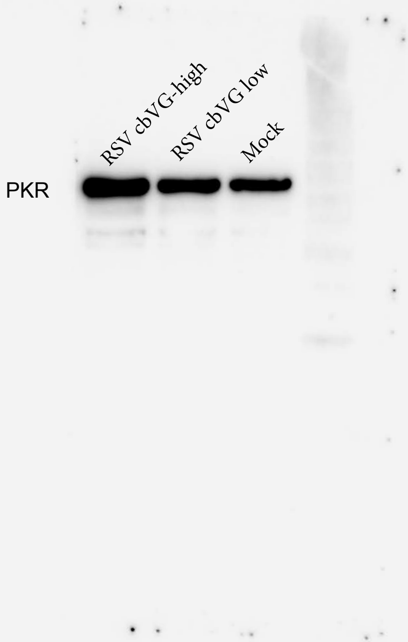

Fig 4A

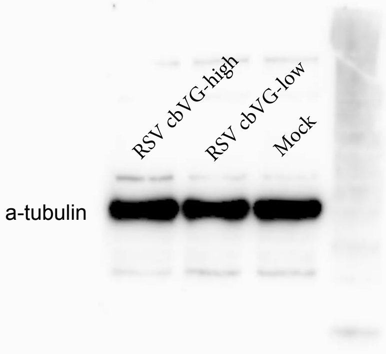

Fig 4B

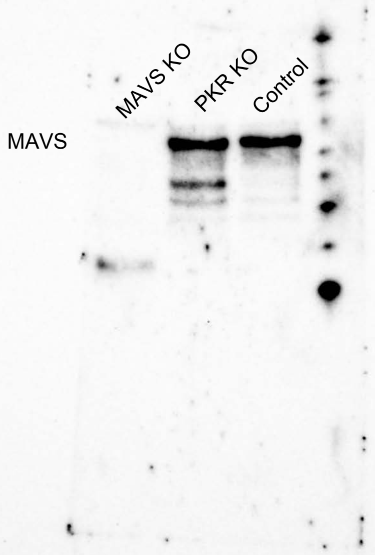

Fig 4B

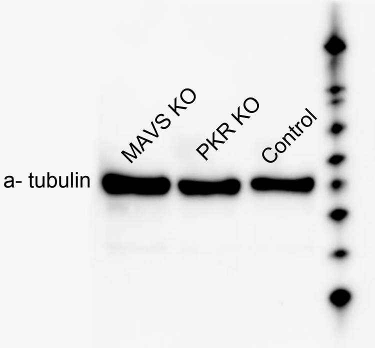

Fig 4B

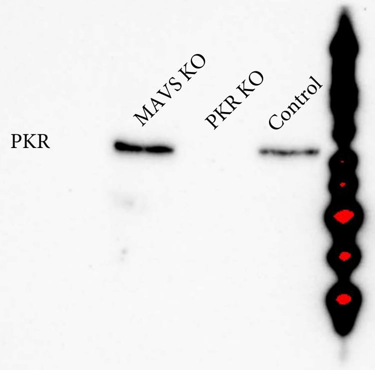

Fig 5A

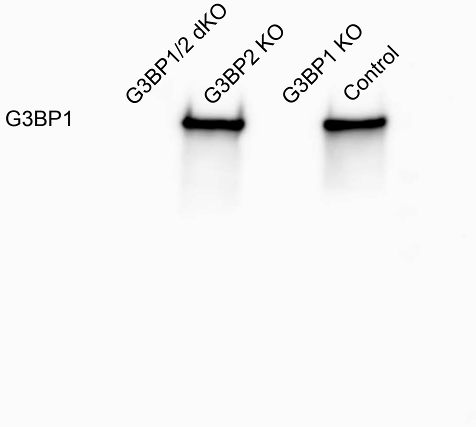

Fig 5A

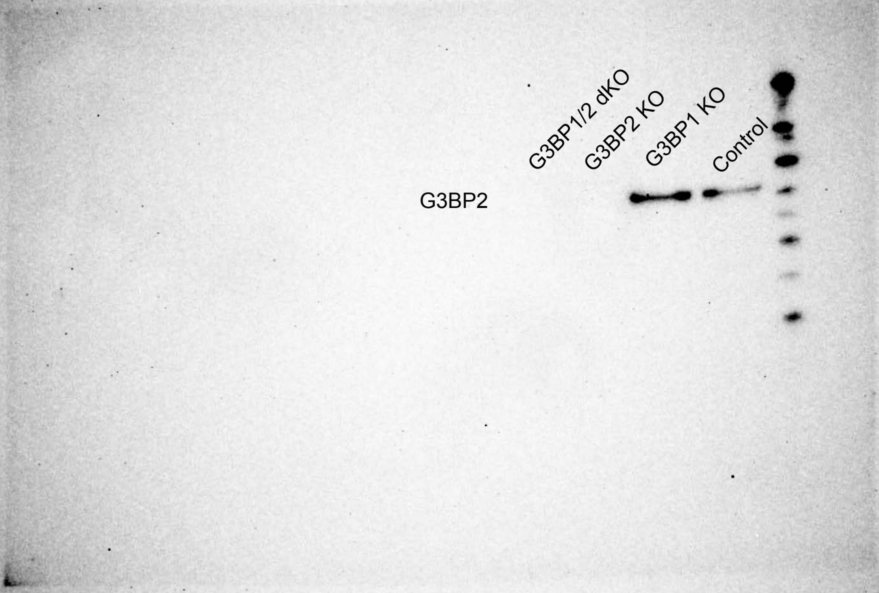

Fig 5A

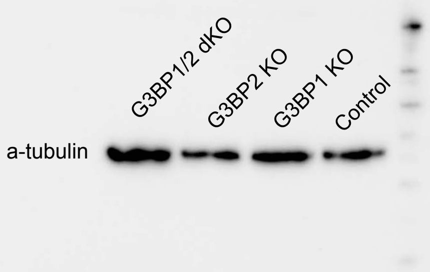

Fig 6E

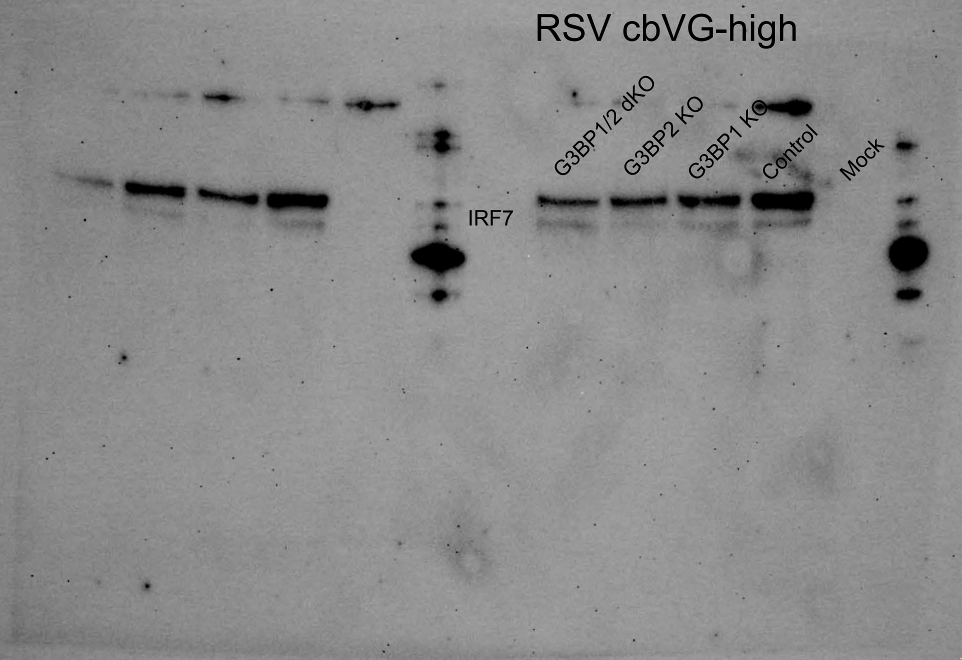

Fig 6E

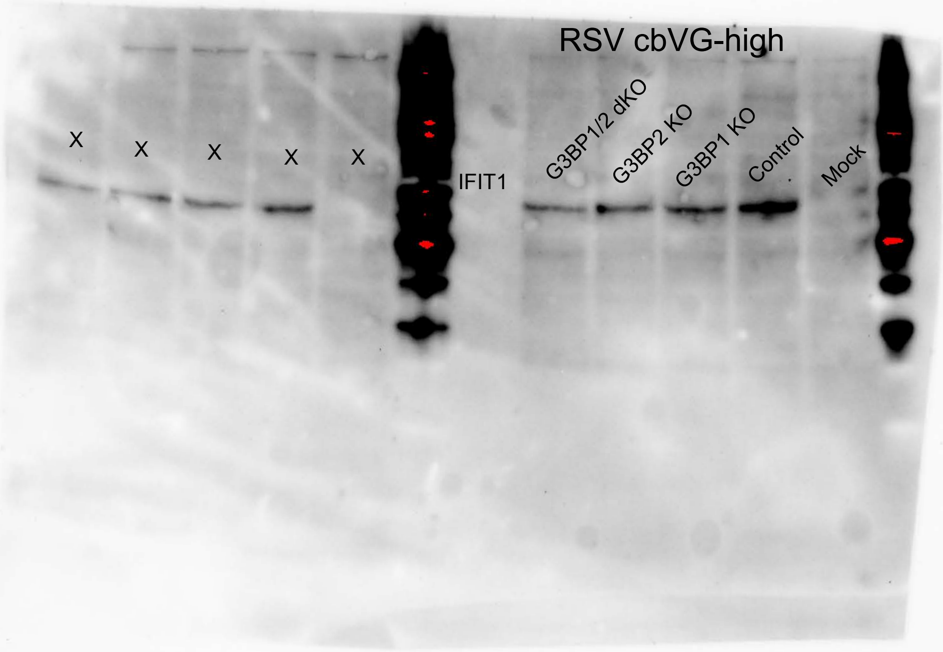
RSV cbVG-high
X
X
X
X
X
IFIT1
G3BP1/2 dKO
G3BP2 KO
G3BP1 KO
Control
Mock

Fig 6E

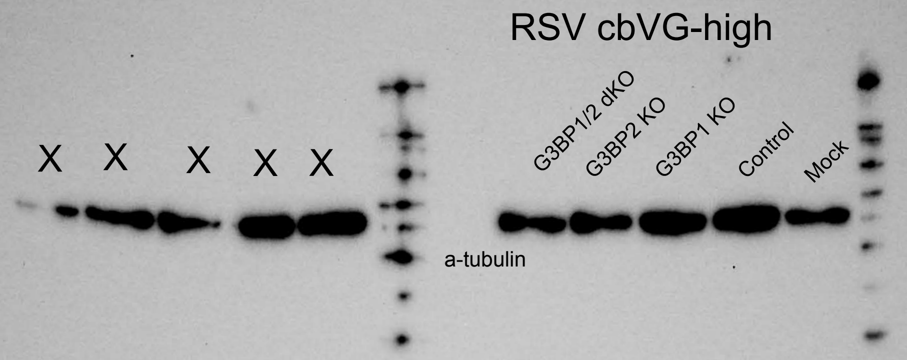
RSV cbVG-high
X
X
X
X
X
G3BP1/2 dKO
G3BP2 KO
G3BP1 KO
Control
Mock
a-tubulin

Fig 6E

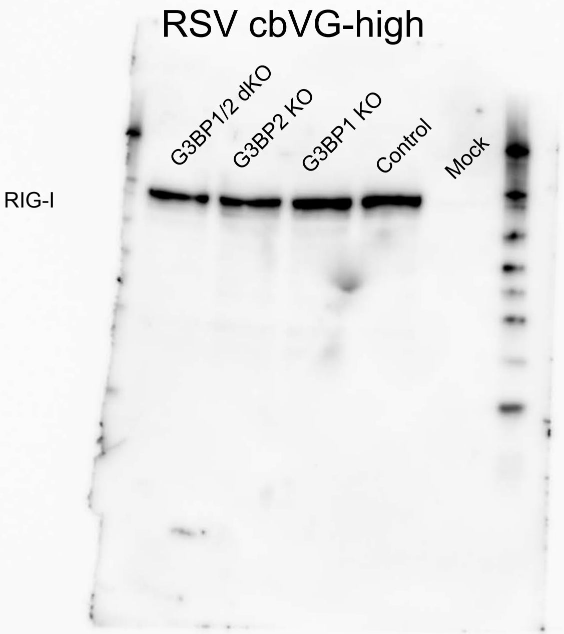

Fig 6E

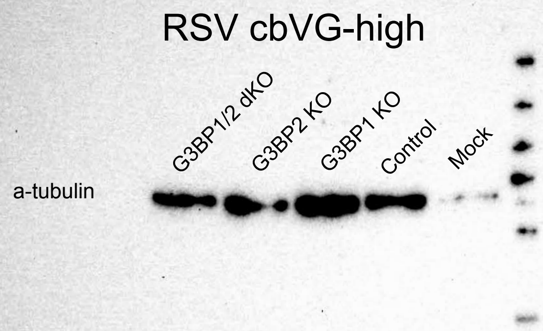
RSV cbVG-high
G3BP1/2 dKO
G3BP2 KO
G3BP1 KO
Control
Mock
a-tubulin

Fig 6H

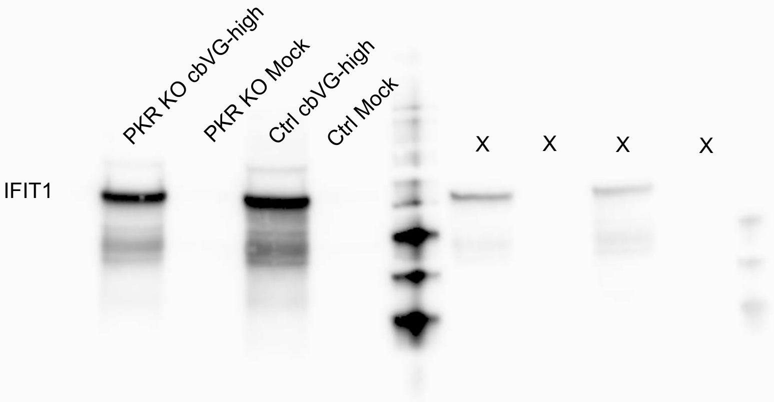
PKR KO cbVG-high
PKR KO Mock
Ctrl cbVG-high
Ctrl Mock
X
X
X
X
IFIT1

Fig 6H

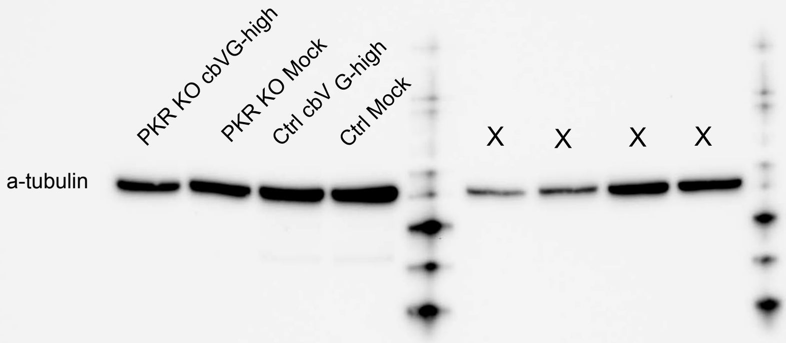
PKR KO cbVG-high
PKR KO Mock
Ctrl cbV G-high
Ctrl Mock
X
X
X
X
a-tubulin

Fig S1B

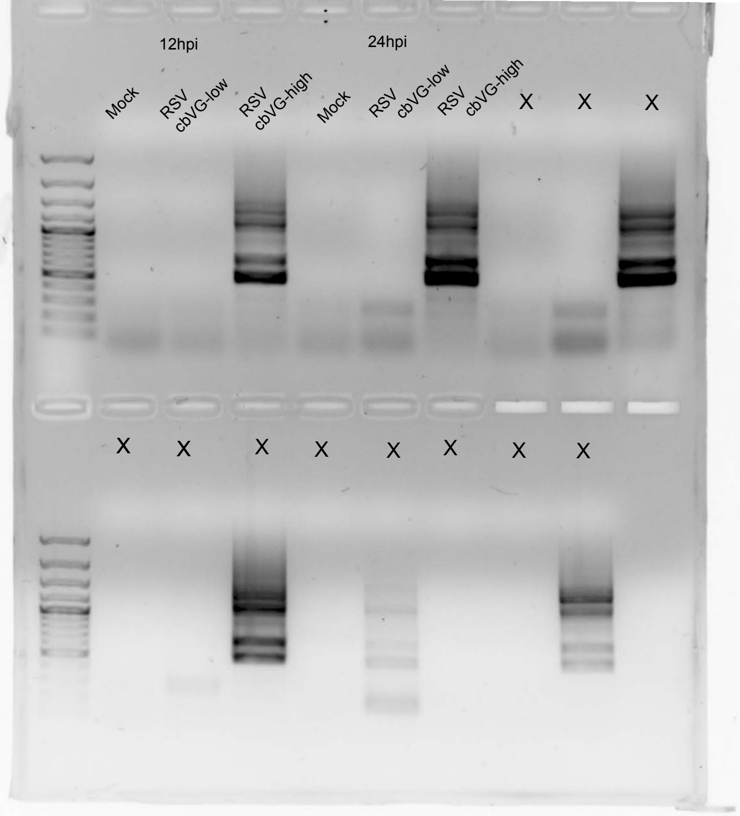
12hpi
24hpi
Mock
RSV cbVG-low
RSV cbVG-high
Mock
RSV cbVG-low
RSV cbVG-high
X
X
X
X
X
X
X
X
X
X
X

Fig S3A

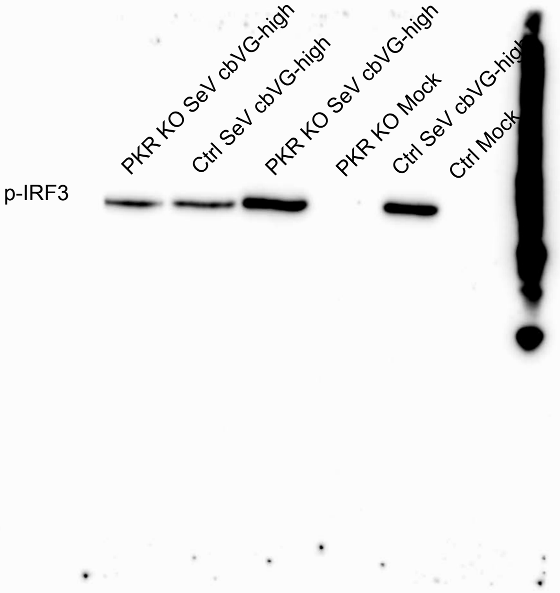
PKR KO SeV cbVG-high
Ctrl SeV cbVG-high
PKR KO SeV cbVG-high
PKR KO Mock
Ctrl SeV cbVG-high
Ctrl Mock
p-IRF3

Fig S3A

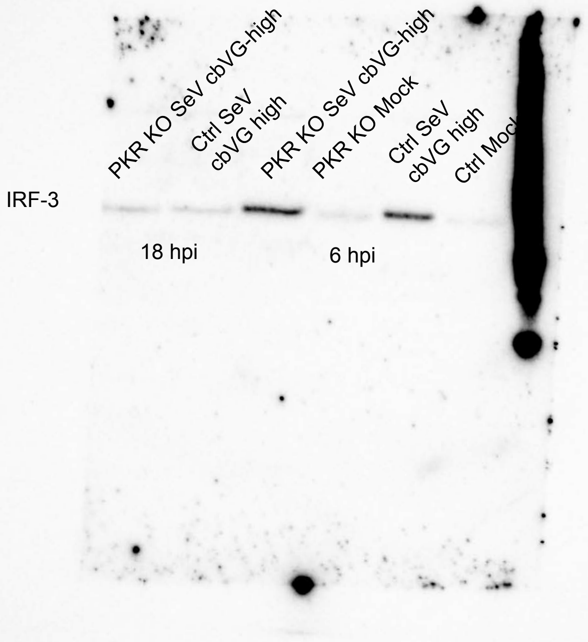
PKR KO SeV cbVG-high
Ctrl SeV cbVG high
PKR KO SeV cbVG-high
PKR KO Mock
Ctrl SeV cbVG high
Ctrl Mock
IRF-3
18 hpi
6 hpi

Fig S3A

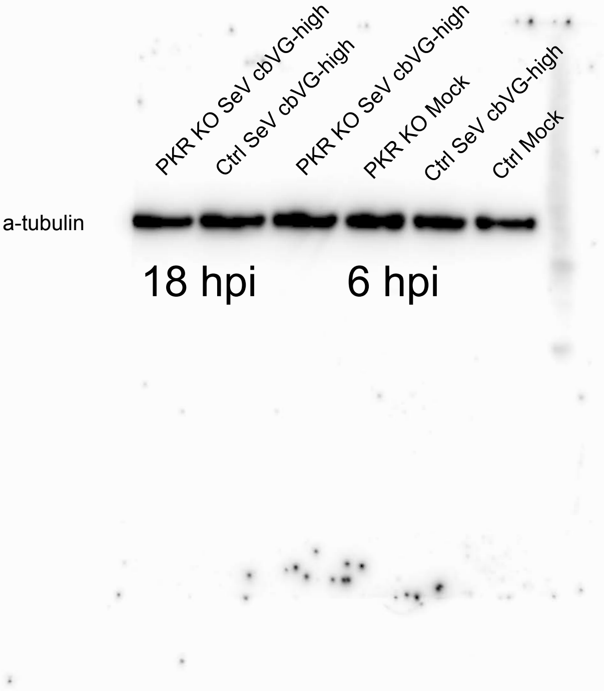
PKR KO SeV cbVG-high
Ctrl SeV cbVG-high
PKR KO SeV cbVG-high
PKR KO Mock
Ctrl SeV cbVG-high
Ctrl Mock
a-tubulin
18 hpi
6 hpi

Fig S3B

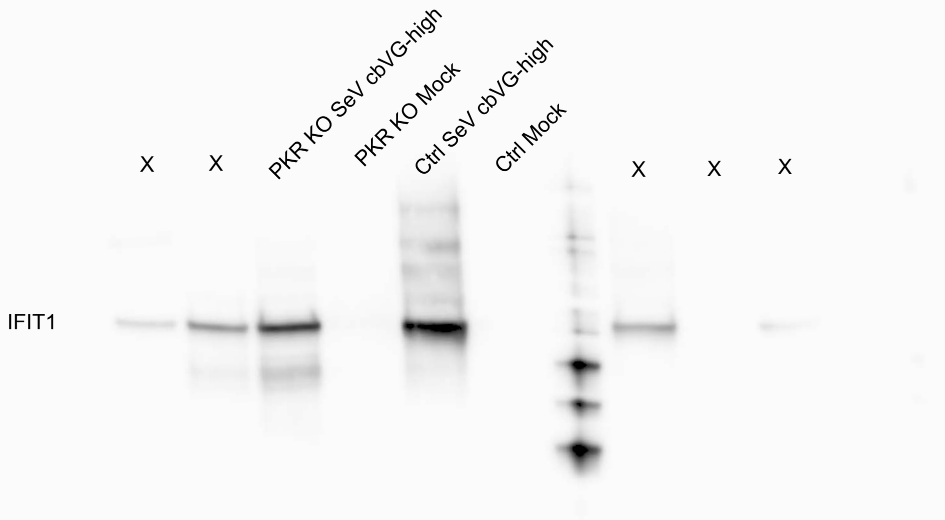

X
X
PKR KO SeV cbVG-high
PKR KO Mock
Ctrl SeV cbVG-high
Ctrl Mock
X
X
X
IFIT1

Fig S3B

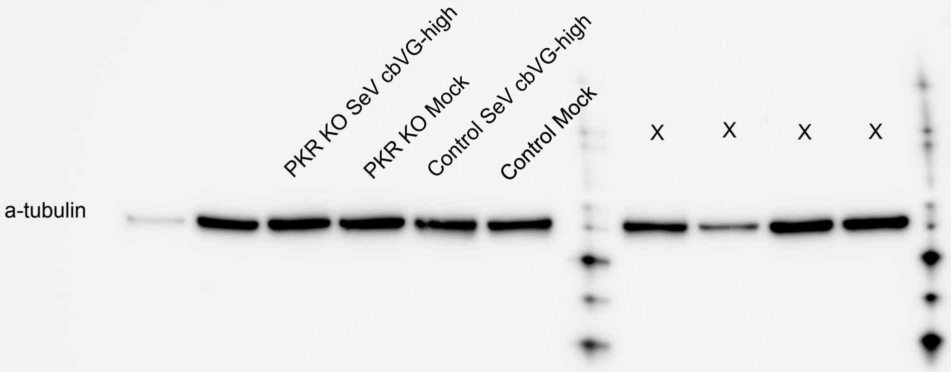
PKR KO SeV cbVG-high
PKR KO Mock
Control SeV cbVG-high
Control Mock
x
x
x
x
a-tubulin
