# Supplementary material for: Copy-back viral genomes induce a cellular stress response that interferes with viral protein expression without affecting antiviral immunity
Source: PLoS Biol. 2023 Nov 20;21(11):e3002381. doi: 10.1371/journal.pbio.3002381 (PMC10695362; doi:10.1371/journal.pbio.3002381)

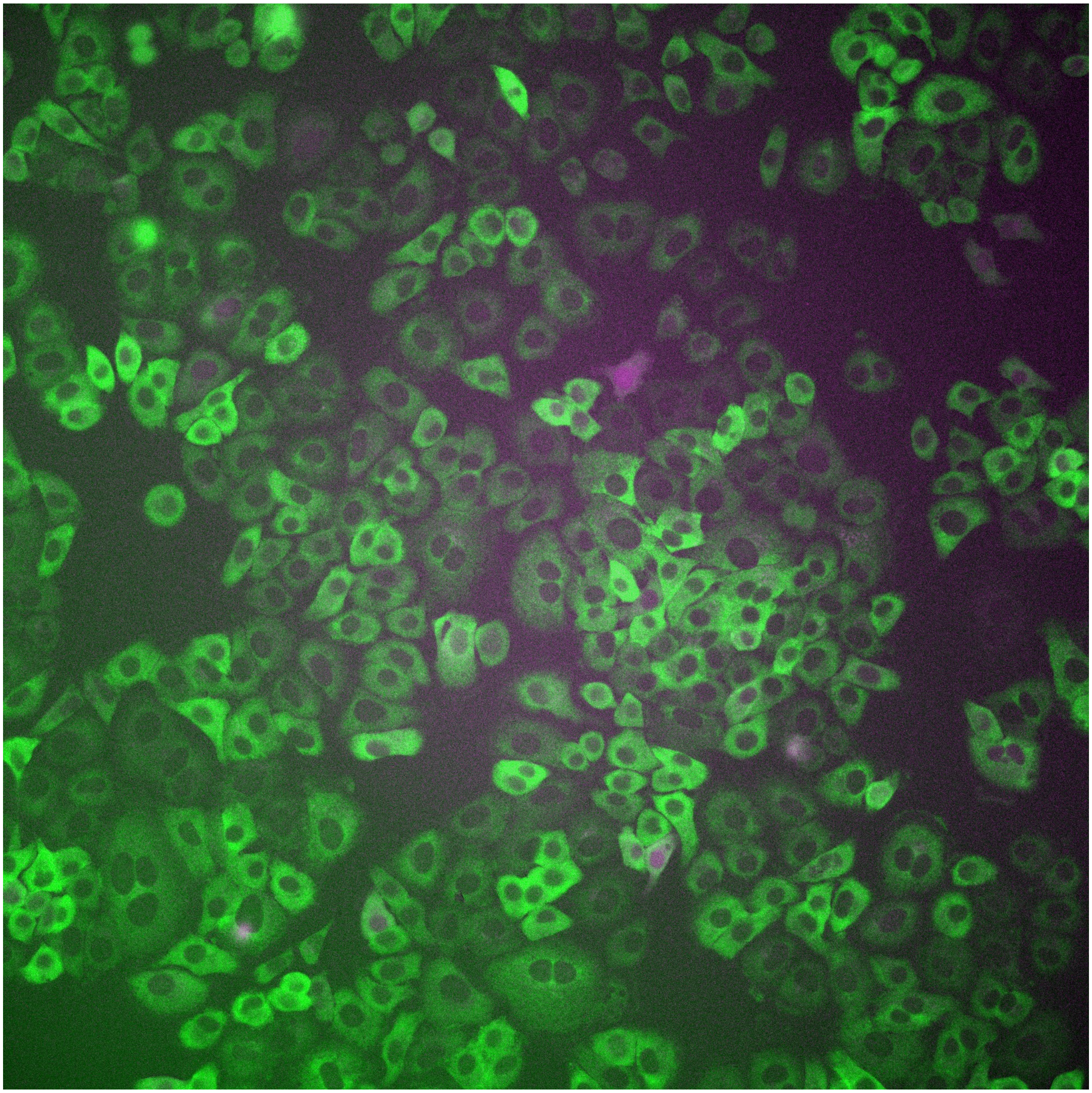

Supplement: Supplementary file 5 [file pbio.3002381.s007.docx]
