# Supplementary material for: Copy-back viral genomes induce a cellular stress response that interferes with viral protein expression without affecting antiviral immunity
Source: PLoS Biol. 2023 Nov 20;21(11):e3002381. doi: 10.1371/journal.pbio.3002381 (PMC10695362; doi:10.1371/journal.pbio.3002381)
Supplement: Attachment — Submitted filename: Response to reviewer_Point by point_rev1_FINAL.pdf [file pbio.3002381.s010.pdf]

Point by Point response:

We thank the reviewers for their constructive revision to our manuscript. We have addressed all the concerns that have resulted in a much-improved report. All revisions are marked in burgundy font in the “marked” copy of the manuscript. Below a point-by-point response to all your concerns:

Reviewer 1: Arbitrating reviewer. Virus-host ribonucleoprotein complexes and innate immunity

The manuscript by Gonzalez Aparicio entitled "Copy-back viral genomes induce a cellular stress response that interferes with viral protein expression without affecting antiviral immunity" links 5' copy-back DVG production upon infections with Sendai or respiratory syncytial virus (RSV) to PKR-dependent formation of canonical SGs complemented by the down-regulation of viral translation. This 5'-copy-back DVG functioning is independent 5'-copy-back functioning in activation of IFN signaling. Presented results are novel and important to understand various mechanisms of virus-host interactions, providing an example where the same pathogen molecular pattern is implemented in several cellular pathways.
However, I have several major remarks:
1) Pfaller et al. J.Virology 2014 paper (PMID: 24155404) should be discussed and cited in the current manuscript. As earlier, using measles virus (another Paramyxoviridae), Pfaller et al. have demonstrated that the deltaC measles virus strain produces dsRNA (copy-back DVG), activates PKR which correlates with SG formation. Thus Plaller et al. study provides initial information on specific RNA molecules -copy-back DVG that trigger PKR signaling and stress granule formation during natural viral infections.

- We acknowledge this oversight. This reference has been added to the manuscript together with new text **on lines 92-98**.

2) The abbreviation of copy-back viral genomes as cbVGs is perturbing. The authors describe 5'-copyback defective viral genomes that are classically abbreviated as copy-back DVG (cbDVGs, by the same and other laboratories: PMID: 30245734, PMID: 26443454, PMID: 29861183, PMID: 34160256, PMID: 34815573 etc) or as copy-back DI RNAs (cb DI-RNA: PMID: 7037195, PMID: 16631220, PMID: 35632848, PMID: 28768856, PMID: 24155404). I believe one of these already established abbreviations should be used throughout the manuscript. Idem for standard genomes (stVG-high) which is simply a full-length genome and used only 3 times in the text of the manuscript. Non-standard viral genomes (nsVGs) are DVG and why they are "hypermutated RNAs": cb DVGs are not hypermutated they just have 5'/3' complementary ends. But their sequences are stable. I addition, Paramyxoviridae respect the so called "rule of six" (PMID: 8392616).

- We are sorry that the reviewer found this change perturbing. This is not the first publication where we have used this new terminology that we believe is more accurate than DVGs, and we think it will solve the issue of multiple parallel names for these virus species (please see PMID: 35658541 and PMID: 36325033). We decided to remove the word defective (from “defective viral genome”) as it creates confusion and the wrong impression that non-standard viral genomes are “defective” and have no function. DVGs was an initial modification from the original term “defective interfering viral genomes” where we removed the word “interfering” several years ago as it wrongly implied a unique function to this large family of non-standard viral genomic forms. We believe that it is now time to eliminate all qualifiers to their name and call these genomes for what they are rather than implying functions (or lack of functions). Furthermore, the other most studied non-standard viral genomes are deletion viral genomes (delVGs), which also do not contain the word “defective” as cited by other groups (Alnaji and Brooke, 2020). So, for sake of accuracy and consistency, we call them cbVGs and delVGs, respectively. stVGs is the natural abbreviation for standard viral genomes as they compare to non-standard viral genomes (nsVGs).
- We are sorry, but we do not understand the comment about “hypermutated RNAs” as we don’t believe cbVGs are described like this at any point in the manuscript.

3) IF microscopy images for all figure (except supplementary figures) are difficult to view. Other main figures, like Figure 6 are also unreadable. Figure with a better resolution should be provided. For many central

(Figs. 3C, 3D, 5C, 5D, 7, and supplementary S2, S4, S5) figures quantification analyses (ex. % infected SG positive cells) should be included. I believe that the information on % non-infected SG positive cells is also important to add.

- We apologize for the lost of resolution on the figures. We think this should not be an issue now.

- Regarding the request for quantifications, we will address these next for the individual figures in question:
  - For Figures 3C and 3D the conclusions made are based on **qualitative differences** in SG vs RLBs as is standard in the field and previously described by Burke et al. (https://doi.org/10.1074/jbc.RA119.011638). These qualitative differences (mostly size and "roundness" the subcellular granules) are extremely difficult to accurately quantified with imaging tools available to us. We have included in the figure controls (poly IC) that serves as the gold standard reference for RLBs for comparison.

  - Figures 5C and 5D show either presence or absence of stress granules in the single knockouts and the double knockouts. We do not see any SG positive cell in the G3BP1/2 dKO (**0% positivity**). This same complete lack of SG in the absence of both G3BP1 and 2 was recently reported by Paget et al. (https://doi.org/10.1016/j.molcel.2023.03.010). We have added this information to the **legend of Figure 5**.

  - In the case of figure 7, S2 and S5, conclusions from these figures are qualitative and based on the presence or absence of SG. **Figure 8 quantitatively addresses these observations** based on the translation inhibition observed in SG positive cells.

  - Quantification is included in Fig S4

  - In all the experiments performed, SG positive cells were always infected albeit having low levels of viral protein due to the translation inhibition we demonstrate occurs in these cells (Figure 8B, C). Hence 100% of the SG+ cells show viral NP. We have now added this information to the text **on lines 138-139.**

4) Western blots should contain information on number of biological replicates and quantification should be added when need (Figs 4A, 6H, S3).

- We have added information of number of replicates in the figure legends and added inverted intensity quantifications for the figures mentioned above.

5) In the abstract part, more careful communication of the observed results with SG formation and antiviral response should be considered as "SG positive cells are only 10 to 20% of the infected population at a given time,.." and " Although we cannot rule out that a reduction on ISG protein expression occurs specifically in SG positive cells, this potential reduction does not affect the global immune antiviral response during the infection,..".

- We have attempted to reflect these facts better in the abstract by adding the word "global" to the effect conclusion. Given the constrains in length of the abstract we believe this modification makes the point that we further develop in subsequent sections of the manuscript.

Minor

1) Additional read out "as a proxy for virus replication" (using another viral RNA or intergenic region could be considered validating the results presented on Figures 4C, 5B, and S1C.

- We understand the reviewer perspective. However, we believe that viral mRNA expression is a sensitive proxy for active virus replication and sufficient to make the points of these figures. We show viral protein expression (NP and F) in the rest of the manuscript as additional method to evaluate active infection. In

addition, we and others have published in several occasions that in this particular system (A549 infected cells 6-24 hpi) viral mRNA reflects virus infectious particles accurately. An example of a comparison of viral mRNA and infectious particles for the related virus Sev can be seen in https://doi.org/10.1371/journal.ppat.1005122 and https://doi.org/10.1371/journal.ppat.1003703

2) Short title on the first page is : "Stress responses during pneumovirus and paramyxovirus infections" and on line 13: "Stress responses during RNA virus infections". Which one is correct?

- This comment has been resolved to keep consistent short title.

3) For Fig/8 the figure legend is missing, and the Fig.8D is unreadable!

  - We double-checked that figure 8 has the legend. We increased the size of Figure 8D to make it more legible.

4) Several titles in materials and methods are written in blue.

- This comment has been resolved.

5) Line 563 S3 or S1?

- This comment has been resolved.

6) On line 383 (E) should be (H). I would suggest to add additional replicates to Figure 6F.

- We have changed E to H.

- We have added triplicates for this figure as it is sufficient to perform statistical analysis and to stay consistent with the rest of the quantifications present in the manuscript, which also contain triplicates. We have modified the graphs to differentiate each point belonging to the same experimental replicate. Data points in each condition from the same biological replicate have the same symbols.
